# Different Types of Family Support as a Moderator in the Mental Health of Older Adults with Dementia

**DOI:** 10.1093/geroni/igaf122.2577

**Published:** 2025-12-31

**Authors:** Wan-Ling Hsu, SangNam Ahn

**Affiliations:** Saint Louis University, St. Louis, Missouri, United States; Saint Louis University, St. Louis, Missouri, United States

## Abstract

Dementia impacts millions in the U.S., with family caregivers, particularly adult children and siblings, providing essential emotional support that can protect mental well-being. However, the impact of different types of family support on dementia-related mental health remains unclear. This study analyzed data from the 2016, 2018, and 2020 waves of the Health and Retirement Study, involving 14,140 U.S. participants aged 50 and older. Dementia was identified through self-reported or proxy reports. Depression was assessed using the Center for Epidemiologic Studies Depression Scale (CESD-8), and loneliness with a three-item UCLA Loneliness Scale. Family support was assessed by the number of living children and siblings. Weighted linear regression examined the association between dementia, family support, and mental health. The sample included 3,579 participants, with 562 (15.7%) having dementia. CESD-8 scores were higher among participants with dementia (+1.98), females (+0.23), non-white participants (+0.38), and those separated/divorced (+0.50) or widowed (+0.28), while those with higher education scored 0.35 points lower. A significant interaction was found between dementia and family support from children, with participants having more children reporting lower CESD-8 scores compared to those without children. On the loneliness scale, younger participants (-0.004) and those with higher education (-0.05) scored lower, while separated/divorced (+0.16) and widowed participants (+0.18) scored higher. No significant interactions were found between dementia and family support on loneliness. Enhancing family support from adult children could reduce depression symptomatology in dementia, offering a valuable approach for policymakers and healthcare providers.

